# Association of time-to-treatment with outcomes of *Pneumocystis* pneumonia with respiratory failure in HIV-negative patients

**DOI:** 10.1186/s12931-019-1188-6

**Published:** 2019-09-26

**Authors:** Ryoung-Eun Ko, Soo Jin Na, Kyungmin Huh, Gee Young Suh, Kyeongman Jeon

**Affiliations:** 1 Department of Critical Care Medicine, Samsung Medical Center, Sungkyunkwan University School of Medicine, 81 Irwon-ro, Gangnam-gu, Seoul, 06351 Republic of Korea; 2 Division of Infectious Diseases, Department of Medicine, Samsung Medical Center, Sungkyunkwan University School of Medicine, Seoul, Republic of Korea; 3 Division of Pulmonary and Critical Care Medicine, Department of Medicine, Samsung Medical Center, Sungkyunkwan University School of Medicine, 81 Irwon-ro, Gangnam-gu, Seoul, 06351 Republic of Korea

**Keywords:** *Pneumocystis* pneumonia, HIV seronegativity, Respiratory insufficiency, Time-to-treatment, Treatment outcome

## Abstract

**Background:**

The prevalence of *pneumocystis* pneumonia (PCP) and associated hypoxic respiratory failure is increasing in human immunodeficiency virus (HIV)-negative patients. However, no prior studies have evaluated the effect of early anti-PCP treatment on clinical outcomes in HIV-negative patient with severe PCP. Therefore, this study investigated the association between the time to anti-PCP treatment and the clinical outcomes in HIV-negative patients with PCP who presented with hypoxemic respiratory failure.

**Methods:**

A retrospective observational study was performed involving 51 HIV-negative patients with PCP who presented in respiratory failure and were admitted to the intensive care unit between October 2005 and July 2018. A logistic regression model was used to adjust for potential confounding factors in the association between the time to anti-PCP treatment and in-hospital mortality.

**Results:**

All patients were treated with appropriate anti-PCP treatment, primarily involving trimethoprim/sulfamethoxazole. The median time to anti-PCP treatment was 58.0 (28.0–97.8) hours. Thirty-one (60.8%) patients were treated empirically prior to confirmation of the microbiological diagnosis. However, the hospital mortality rates were not associated with increasing quartiles of time until anti-PCP treatment (*P* = 0.818, test for trend). In addition, hospital mortality of patients received early empiric treatment was not better than those of patients received definitive treatment after microbiologic diagnosis (48.4% vs. 40.0%, *P* = 0.765). In a multiple logistic regression model, the time to anti-PCP treatment was not associated with increased mortality. However, age (adjusted OR 1.07, 95% CI 1.01–1.14) and failure to initial treatment (adjusted OR 13.03, 95% CI 2.34–72.65) were independently associated with increased mortality.

**Conclusions:**

There was no association between the time to anti-PCP treatment and treatment outcomes in HIV-negative patients with PCP who presented in hypoxemic respiratory failure.

## Background

*Pneumocystis* pneumonia (PCP), a pulmonary infection caused by *Pneumocystis jirovecii*, remains one of the most prevalent opportunistic infections in immunocompromised patients [1–3]. The development of highly active antiretroviral therapy and effective prophylaxis against PCP have reduced the mortality of PCP in patients with human immunodeficiency virus (HIV) [4, 5]. However, as the numbers of patients receiving immunosuppressive therapy after organ transplantation and antitumor chemotherapy increase, the incidence of PCP in HIV-negative patients has also increased [2, 6–8]. Typically, HIV-positive patients follow a more insidious course [2, 9]. In contrast, HIV-negative patients with PCP present with abrupt-onset hypoxemic respiratory failure [9, 10]. Therefore, it is necessary to treat the infection before complications from respiratory failure arise. However, early treatment is sometimes difficult, as the diagnosis of PCP is mostly a clinical one in critical care settings [9].

Over the past several decades, trimethoprim/sulfamethoxazole (TMP/SMX) remains the drug of choice for PCP [2, 9, 11]. Because of the high mortality rate in HIV-negative patients, it has been suggested that anti-PCP treatment should be started empirically in patients who are suspected to have moderate to severe PCP [12]. However, TMP/SMX induces several side effects, including granulocytopenia, skin eruptions, hepatotoxicity, and renal toxicity. Using TMP/SMX inappropriately can also allow resistant strains to develop [13]. There is a lot of literature regarding PCP in HIV-negative patients; however, previous studies have focused on patient factors and laboratory findings associated with clinical outcomes [14, 15]. Few have investigated the delayed initiation of anti-PCP treatment therapy in HIV-negative patients compared to HIV-positive patients [16, 17]. No prior study has evaluated the effect of early anti-PCP treatment on the clinical outcomes in HIV-negative patients with severe PCP.

Therefore, this study investigated the association between the time to anti-PCP treatment and the clinical outcomes in HIV-negative patients with PCP who presented with hypoxemic respiratory failure.

## Methods

We retrospectively reviewed the medical records of all consecutive patients with PCP who were admitted to the medical intensive care unit (ICU) for respiratory failure at Samsung Medical Center (a 1979-bed, university-affiliated, tertiary referral hospital in Seoul, South Korea) between October 2005 and July 2018. The study was approved by the Institutional Review Board of Samsung Medical Center. Informed consent was waived because of the retrospective observational nature of the study. All patient records and data were anonymized and de-identified prior to analysis.

### Study population

**Fig. 1:**
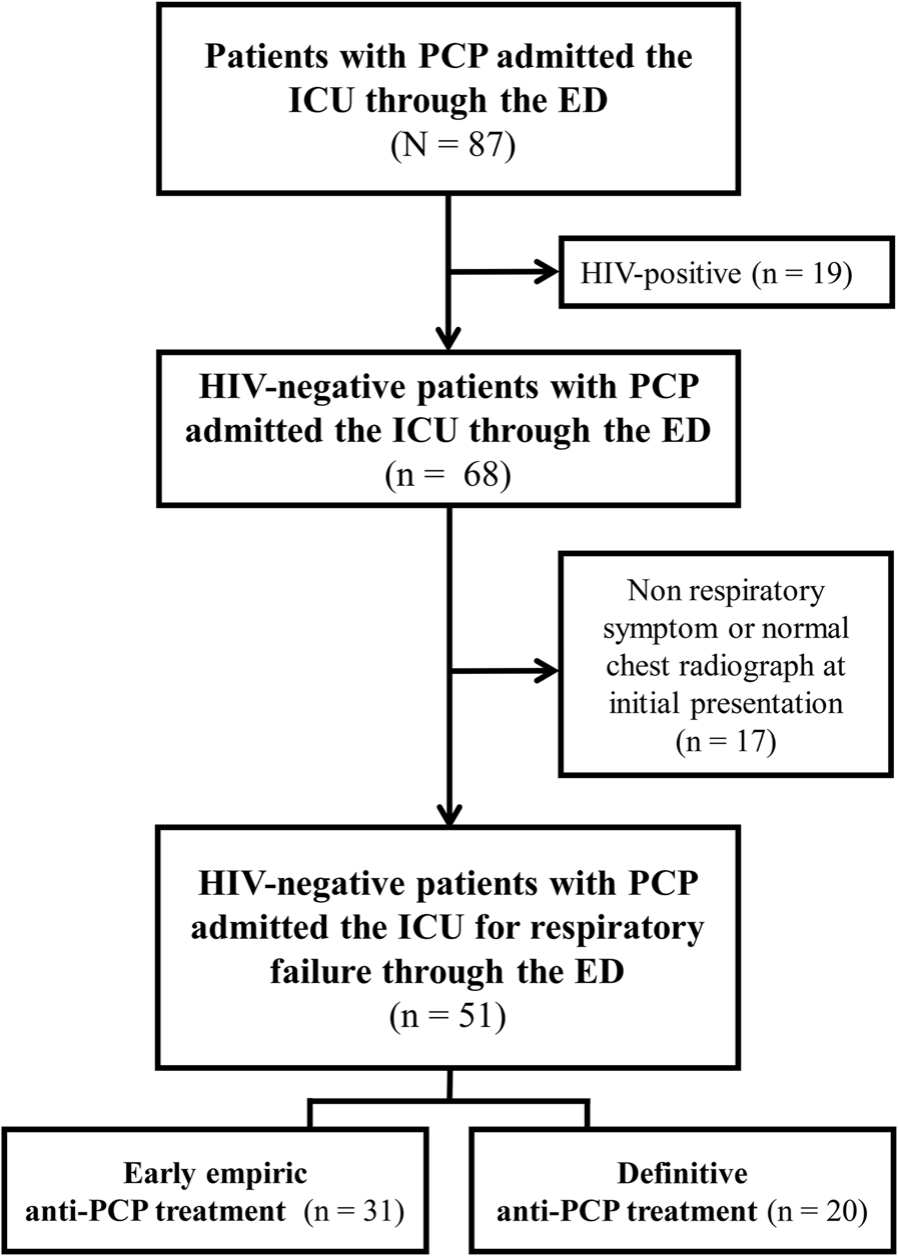
Study flow diagram. PCP, *Pneumocystis* pneumonia; ICU, intensive care unit; ED, emergency department

All consecutive patients older than 20 years who were admitted to the medical ICU through the emergency department (ED) for acute respiratory failure were screened for inclusion (Fig. 1). Some of the clinical data from patients who were enrolled between 2005 and 2011 were also included in the previous study [18]. Patients were included if they had a microbiologically confirmed diagnosis of PCP and required respiratory support with mechanical ventilation including non-invasive ventilation or high-flow nasal cannula for respiratory failure. Patients were excluded if they had a positive HIV antibody test. Finally, patients who had neither respiratory symptom nor abnormal finding on chest radiographs at initial presentation were excluded from the study. In patients who had multiple admissions for acute respiratory failure due to PCP during the study period, only the first ICU admission was evaluated.

The diagnosis of PCP was based on clinical symptoms and the presence of a new infiltration on chest radiograph, along with morphological identification of the organism in bronchoalveolar lavage (BAL) fluid or lung tissue obtained by transbronchial lung biopsy (TBLB). The BAL fluid samples were stained using Gram and Ziehl-Neelsen methods and then cultured for bacteria, mycobacteria, and fungi. Multiplex nested polymerase chain reaction (PCR) assays were used to detect the following: influenza viruses A and B; parainfluenza viruses 1, 2, and 3; respiratory syncytial virus; and adenovirus [19]. Quantitative real-time PCR was used to measure the cytomegalovirus (CMV) deoxyribonucleic acid (DNA) in the BAL fluid [20]. Microbiological identification of *P.jirovecii* was confirmed by documenting the organism with Wright Giemsa or Gram-Weigert stain, or the cyst with the Gomori methenamine silver or calcofluor white stain [2].

### Data collection

The following data were extracted from the medical records: general demographic information, underlying diseases, medications used during the previous month, PCP prophylaxis, need for renal replacement therapy, need for vasopressor support, anti-PCP medication, and newly developed organ failure during the ICU stay. We also collected the following laboratory data from the medical records: the white blood cell count, absolute neutrophil count, absolute lymphocyte count, albumin, C-reactive protein, arterial partial pressure of oxygen (PaO_2_)/fraction of inspired oxygen (FiO_2_) ratio (PF ratio), and alveolar-arterial oxygen gradient [D(A-a)O_2_]. The severity of illness was assessed using the Simplified Acute Physiology Score 3 (SAPS 3) and Sequential Organ Failure Assessment (SOFA) score [21, 22]. Finally, we studied the clinical outcomes, including the length of ICU stay, the length of hospital stay, and ICU and in-hospital mortality.

The time to anti-PCP treatment interval was defined as the number of hours from the time of emergency room arrival to the initial administration of anti-*Pneumocystis* antibiotics. We classified patients into two groups according to the timing of initial anti-PCP treatment before or after microbiologic diagnosis [23]: early empiric treatment group and definitive treatment group. The doses of corticosteroids used for immunosuppression and the adjunctive therapy for PCP were expressed as the prednisolone-equivalent doses [24]. The adjunctive corticosteroid therapy was defined as that started within 72 h of initiating the specific anti-PCP treatment (consisting of at least 40 mg prednisone twice daily for 5 days regardless of the subsequent tapering schedule), and the use of corticosteroids before the onset of PCP [25]. The failure of anti-PCP treatment was defined as follows: (1) progressive clinical deterioration as demonstrated by the inability to maintain a stable PaO_2_ despite an increase in FiO_2_; and (2) progressive deterioration of vital signs with an increased FiO_2_ requirement despite 7 days of appropriate therapy [11, 26]. Breakthrough PCP infection was defined as that diagnosed in a patient receiving prophylactic agents with known activity against *P. jirovecii* for at least 7 days prior to the diagnosis [27].

### Statistical analysis

All data are presented as medians and interquartile range (IQR) for continuous variables, and as numbers and percentages for categorical variables. Continuous variables were analyzed using the Mann-Whitney-Wilcoxon U test. Categorical variables were analyzed using the Pearson χ2 test or Fisher exact test. The Mantel–Haentzel trend test was used to examine the trends in the hospital mortality rate across the time quartiles to anti-PCP treatment [28]. To adjust for potential confouding factors in the association between the time to anti-PCP treatment and in-hospital mortality, multiple logistic regression analysis was used. Finally, we compared baseline characteristics and clinical outcomes between early empiric treatment group and definitive treatment group. Probabilities of survival after anti-PCP treatment for each group were estimated by Kaplan-Meier method and compared by the log-rank test. Variables with a *P*-value less than 0.2 on univariate analyses [29], as well as a priori variables of age and sex were entered into a multiple logistic regression model in which in-hospital mortality was the outcome variable of interest. To reduce the risk of multicollinearity, one closely correlated variable was a candidate for inclusion in the final model. Data are presented as adjusted odds ratios (OR) with 95% confidence intervals (CI). All of the tests were two-sided. *P* values < 0.05 were considered statistically significant. All analyses were performed using SPSS for Windows (ver. 23.0; IBM Corp., Armonk, NY, USA).

## Results

### Study population

During the study period, a total of 117 patients with PCP were admitted to the ICU for respiratory support. Nineteen of these patients were excluded from the study because they had HIV infections. After excluding patients who were initially admitted to general ward from outpatient care with other cause (*n* = 29), those who were transfer to our hospital (*n* = 1), and those with an initial chief complaint that was unrelated to respiratory symptoms (*n* = 17), 51 patients with acute hypoxemic respiratory failure and a new infiltration on chest radiograph were included.

The patients’ baseline characteristics are summarized in Table 1. The median patient age was 52.0 (IQR 40.5–66.0) years. Thirty-five (68.6%) patients were men. The main underlying conditions associated with the development of PCP included malignancies (*n* = 28, 54.9%) and history of solid organ transplant (*n* = 18, 35.3%). All of the patients were immunosuppressed before developing PCP. Twenty-two (43.1%) patients received systemic steroids. The median prednisolone-equivalent dose was 16.1 (6.2–34.7) mg/day in patients who received systemic steroids. Two (3.9%) patients had a history of receiving prophylactic TMP/SMZ.

**Table 1:** Baseline patient characteristics

Characteristics	No. of patients (%) or median (IQR)
Age, years	52.0 (40.5–66.0)
Sex, male	35 (68.6)
Underlying disease	
Malignancy	28 (54.9)
Hematologic	21
Solid	7
Solid organ transplant	18 (35.3)
Kidney	11
Liver	4
Heart	2
Lung	1
Other^a^	5 (9.8)
Immunosuppressive agents used, previous month^b^	
Steroid only	18 (35.3)
Chemotherapy only	29 (56.9)
Steroid with chemotherapy	4 (7.8)
Prednisolone-equivalent dose, mg, if steroid used	16.1 (6.2–34.7)
Pneumocystis prophylaxis	2 (3.9)
Chest radiography findings	
Pleural effusion	11 (21.6)
Radiographic pulmonary pattern	
Focal or diffuse alveolar pattern	24 (47.1)
Focal or diffuse interstitial pattern	5 (9.8)
Focal or diffuse alveolar-interstitial pattern	22 (43.1)
Laboratory findings on ICU admission	
WBC blood cells,/μL	7960.0 (5320.0–12,225.0)
Neutrophils,/μL	5458.0 (3245.0–10,395.0)
Lymphocytes,/μL	580.0 (324.0–1020.0)
Albumin, g/dL	2.8 (2.4–3.3)
CRP, mg/dL	12.4 (8.2–19.6)
PaO_2_/FiO_2_ ratio, mmHg	137.2 (112.8–164.6)
D(A-a)O_2_, mmHg	39.9 (30.8–52.3)
Organ failure	
Shock	12 (23.5)
Renal failure requiring renal replacement therapy	1 (2.0)
Respiratory support on ICU admission day	
Mechanical ventilation	43 (84.3)
High-flow nasal cannula	8 (15.7)
Severity of illness	
SAPS 3	48.0 (37.0–57.5)
SOFA	6.0 (5.0–8.0)

*No.* number, *IQR* interquartile range, *ICU* intensive care unit, *WBC* white blood cell, *CRP* C-reactive protein, *PaO*_*2*_*/FiO*_*2*_ arterial partial pressure of oxygen/fraction of inspired oxygen, *D(A-a)O*_*2*_ alveolar-arterial oxygen gradient, *SAPS 3* Simplified Acute Physiology Score 3, *SOFA* Sequential Organ Failure Assessment

^a^Others include 2 glomerulonephritis, 2 interstitial lung disease, and 1 liver cirrhosis

^b^Chemotherapy includes 4 patients with T-cell immunosuppressant

At the time of ICU admission, all of the patients had hypoxemia requiring mechanical ventilation (*n* = 43, 84.3%) or high-flow nasal cannula support (*n* = 8, 15.7%). The median PF ratio was 137.2 (112.8–164.6) mmHg. The D(A-a)O_2_ was 39.9 (30.8–52.3) mmHg. Twelve (23.5%) patients required vasopressor support and one (2.0%) needed renal replacement therapy. The median SAPS 3 and SOFA scores on ICU admission were 48.0 (37.0–57.5) and 6.0 (5.0–8.0), respectively.

### Diagnosis and treatment of PCP

All of the patients underwent bronchoscopy with BAL ± TBLB within 57.7 (30.2–91.7) hours of the ER visit (Table 2). The diagnosis of PCP was based on microbiological identification of *P. jirovecii* in the BAL fluid (*n* = 44, 86.3%) or lung biopsy specimens (*n* = 14, 27.5%). Seven (13.7%) patients were positive for *P. jirovecii* in both the BAL fluid and pathology specimens. In addition to *P. jirovecii*, other pathogens were simultaneously isolated from respiratory specimens at the time of diagnosis. The most common simultaneously isolated pathogen was CMV in 13 patients (25.5%), bacteria in 12 (23.5%), and viruses other than CMV in 7 (13.7%).

**Table 2:** Diagnosis and treatment of *Pneumocystis* pneumonia and clinical outcomes

Variables	No. of patients (%) or median (IQR)
Microbiological diagnosis	
Bronchoalveolar lavage fluid	39 (76.5)
Lung biopsy specimen^a^	12 (23.5)
Time to anti-PCP treatment, hours	58.0 (28.0–97.8)
Empiric treatment	31 (60.8)
Other pathogens identified from respiratory specimens	
Cytomegalovirus	13 (25.5)
Virus other than cytomegalovirus^b^	7 (13.7)
Bacteria	11 (21.6)
MRSA	5
*Acinetobacter*	4
*Pseudomonas*	2
Initial treatment regimen	
Trimethoprim/sulfamethoxazole	51 (100.0)
Adjunctive corticosteroid treatment	50 (98.0)
Failure to initial treatment	22 (43.1)

*No.* number, *IQR* interquartile range, *PCP Pneumocystis* pneumonia, *MRSA* methicillin-resistant *Staphylococcus aureus*

^a^7 patients had positive results for the presence of *P.jirovecii* in both BAL fluid and biopsy specimens

^b^Viruses other than cytomegalovirus included rhinovirus (*n* = 3), coronavirus (*n* = 2), and rhinovirus (*n* = 2)

**Fig. 2:**
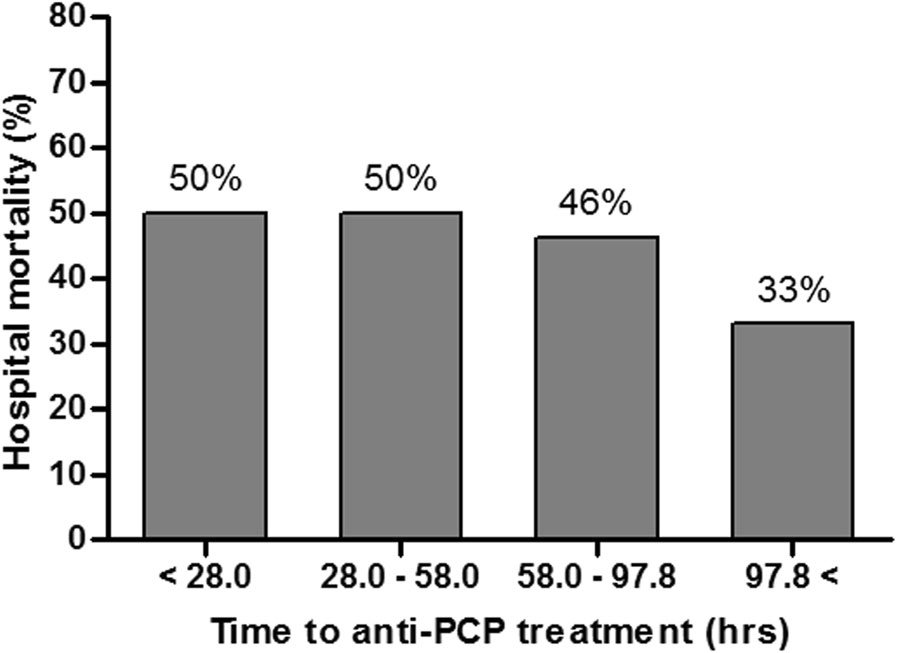
Trends in hospital mortality rate according to time to anti-PCP treatment in quartiles. PCP, *Pneumocystis* pneumonia

All of the patients were treated with appropriate anti-PCP treatment, primarily involving TMP (20 mg/kg/day)/SMZ (100 mg/kg/day). Thirty-one (60.8%) patients were treated with TMP/SMZ empirically prior to confirmation of the microbiological diagnosis. The median time to anti-PCP treatment was 58.0 (28.0–97.8) hours. Baseline characteristics and the hospital mortality rates according to the time to anti-PCP treatment in quartiles are presented in Additional file 1: Table S1 and Fig. 2, respectively. Interestingly, the hospital mortality rates were not associated with increasing quartiles of time to anti-PCP treatment (*P* = 0.818, test for trend).

All of the patients except one (98.0%) were treated with adjunctive corticosteroids. One patient had an adverse reaction to TMP/SMZ involving an electrolyte imbalance after 7 days of use. After excluding this patient, the initial treatment response was assessed on day 7. Of the 50 patients, 20 (40.0%) did not respond to the initial treatment with TMP/SMZ. These 20 patients were subsequently treated with clindamycin-primaquine (*n* = 14) or pentamidine (*n* = 6) as salvage therapy for PCP.

During their ICU stays, organ failure newly developed in 33 (64.7%) patients, which included shock requiring vasopressors in 30 patients and acute kidney injury requiring renal replacement therapy in 13 (Table 3). The ICU mortality was 37.3% with a median stay of 15.0 (6.0–29.0) days. Ultimately, 23 (45.1%) patients died while hospitalized. The median hospital stay was 24.0 (15.5–32.5) days.

**Table 3:** Clinical ICU course

Characteristics	Overall (*N* = 51)	Early empiric treatment (*n* = 31)	Definitive treatment (*n* = 20)	*P* value
New development of organ failure^a^				
Shock	30 (58.8)	20 (64.5)	10 (50.0)	0.461
Renal failure requiring renal replacement therapy	13 (25.5)	6 (19.4)	7 (35.0)	0.356
Extracorporeal membrane oxygenation	2 (3.9)	2 (6.5)	0 (0.0)	0.674
Outcomes				
ICU mortality	19 (37.3)	13 (41.9)	6 (30.0)	0.573
Length of stay in ICU (days)	15.0 (6.0–29.0)	18.0 (8.5–30.5)	11.0 (5.0–19.0)	0.162
Hospital mortality	23 (45.1)	15 (48.4)	8 (40.0)	0.765
Length of stay in hospital (days)	24.0 (15.5–32.5)	25.0 (16.0–34.5)	20.5 (15.5–32.0)	0.493

Data are presented as number (percentage) or as median (interquartile range)

*ICU* intensive care units

^a^Two patients developed shock and renal failure requiring both renal replacement therapy and extracorporeal membrane oxygenation support. Eight patients developed both shock and renal failure requiring renal replacement therapy

**Fig. 3:**
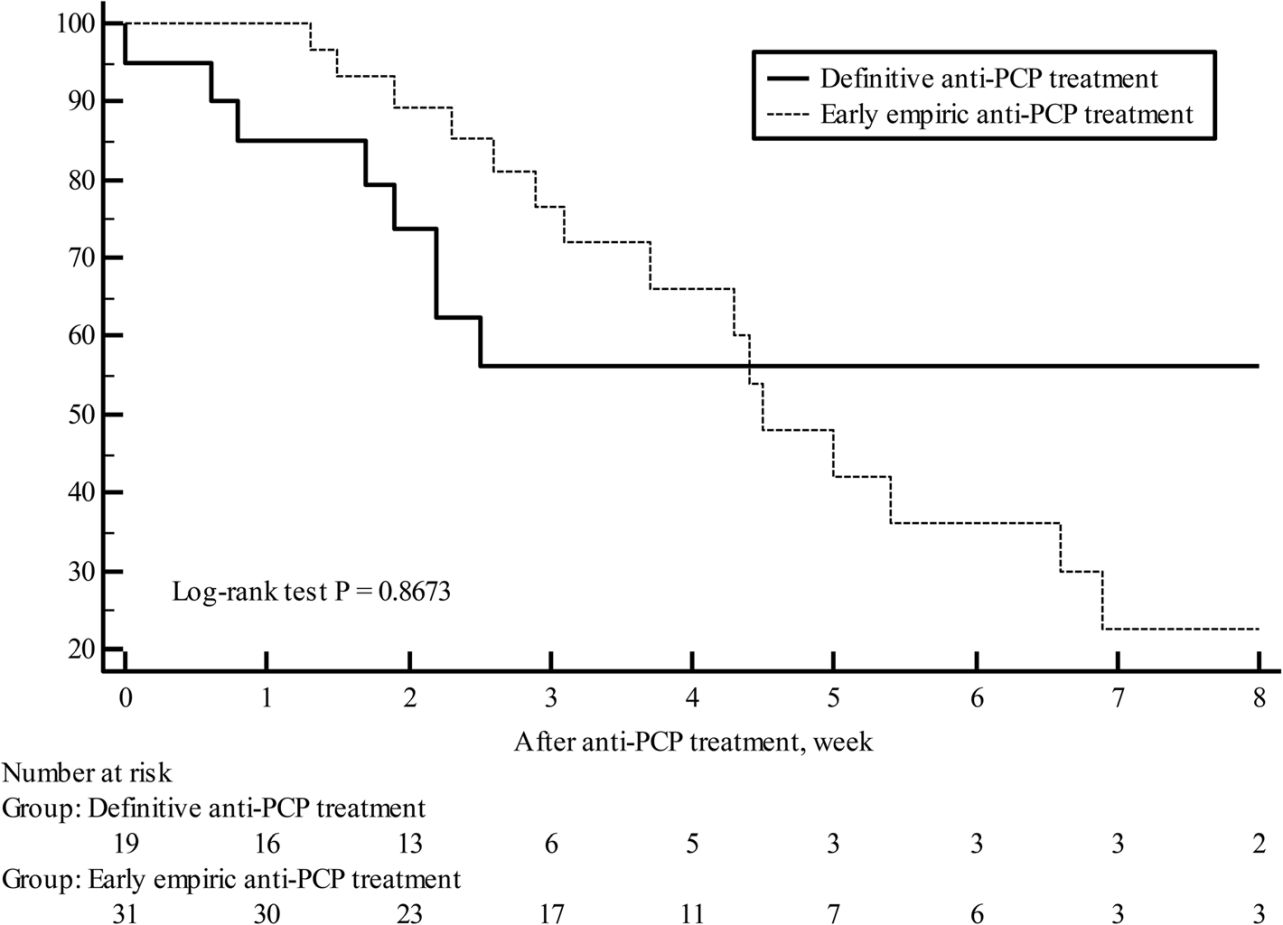
Kaplan-Meier curves of the probability of survival in patients who received early empiric anti-*Pneumocystis* pneumonia (PCP) treatment before microbiologic diagnosis (early empiric treatment group; dotted line) and those who received definitive anti-PCP treatment after microbiologic diagnosis (definitive treatment group; solid line)

The early empiric treatment group contained 31 (60.8%) patients and the definitive treatment group contained 20 (39.2%) patients. There were no significant differences in baseline characteristics, and diagnosis and treatment of PCP, except of the median time to anti-PCP treatment (34.2 h for early empiric treatment group vs. 91.7 h for definitive treatment group, *P* < 0.001) (Additional file 2: Table S2 and Additional file 3: Table S3). In comparison of clinical outcomes between the two groups, there was no significant difference in mortalities and length of stay (Table 3). In addition, early empiric treatment did not significantly affect overall survival (Fig. 3). Although the probability of survival for early empiric treatment group appears to increase in the first few weeks after anti-PCP treatment, this difference is not statistically significant (*P* = 0.8673, log-rank test).

### Factors associated with increased hospital mortality

Univariate comparisons of the clinical characteristics of hospital survivors and non-survivors are presented in Table 4. Compared to survivors, non-survivors were likely to be older (48.0 [34.5–59.0] vs. 64.0 [50.5–69.0], *P* = 0.007), require more mechanical ventilation (67.9% vs. 100%, *P* = 0.009), SAPS 3 score increase (43.0 [30.5–54.5] vs. 56.0 [46.5–58.0], *P* = 0.030), more CMV co-infection (10.7% vs. 43.5%, *P* = 0.011), and failed to initial anti-PCP treatment (21.4% vs. 69.6%, *P* = 0.002). After adjusting for potential confounding factors, age (adjusted OR 1.07, 95% CI 1.01–1.14, *P* = 0.010) and failure to initial treatment (adjusted OR 13.03, 95% CI 2.34–72.65, *P* = 0.005) were independently associated with increased mortality (Table 5). However, the time to anti-PCP treatment was not associated with increased mortality.

**Table 4:** Univariate comparisons of the clinical characteristics of hospital survivors and non-survivors

Characteristics	Survivors (*n* = 28)	Non-survivors (*n* = 23)	*P* value
Age, years	48.0 (34.5–59.0)	64.0 (50.5–69.0)	0.007
Sex, male	17 (60.7)	18 (78.3)	0.298
Underlying disease			0.589
Malignancy	13 (46.4)	15 (65.2)	
Solid organ transplant	12 (42.9)	6 (26.1)	
Others	3 (10.7)	2 (8.7)	
Immunosuppressive agent use, previous month			0.548
Steroid only	9 (32.1)	9 (39.1)	
Chemotherapy only	4 (14.3)	1 (4.3)	
Steroid with chemotherapy	15 (53.6)	13 (56.5)	
Pneumocystis prophylaxis	0 (0.0)	2 (8.7)	0.386
Chest radiography findings			
Pleural effusion	8 (28.6)	3 (13.0)	0.305
Radiographic pulmonary patterns			1.000
Focal or diffuse alveolar pattern	13 (46.4)	11 (47.8)	
Focal or diffuse interstitial pattern	3 (10.7)	2 (8.7)	
Focal or diffuse alveolar-interstitial pattern	12 (42.9)	10 (43.5)	
Laboratory findings on ICU admission	9215.0 (5320.0–12,225.0)	6980.0 (5245.0–12,535.0)	0.757
WBC blood cells, /μL	5591.0 (2880.0–10,395.0)	5458.0 (4235.0–10,960.0)	0.670
Neutrophils, /μL	648.0 (389.5–1278.0)	560.0 (278.0–724.5)	0.244
Lymphocytes, /μL	8.7 (4.0–16.0)	11.9 (5.1–28.8)	0.232
Albumin, g/dL	2.9 (2.5–3.6)	2.8 (2.4–3.0)	0.136
CRP, mg/dL	11.8 (8.4–19.1)	15.0 (7.4–21.8)	0.570
PaO_2_/FiO_2_ ratio, mmHg	147.0 (102.3–170.5)	131.2 (116.8–160.6)	0.619
D(A-a)O_2_, mmHg	38.5 (30.6–51.5)	41.6 (31.1–54.0)	0.771
Organ failure on ICU admission			
Shock	7 (25.0)	5 (21.7)	1.000
Renal failure requiring renal replacement therapy	0 (0.0)	1 (4.3)	0.921
Respiratory support on ICU admission day			0.016
Mechanical ventilation	20 (71.4)	23 (100.0)	
High-flow nasal cannula	8 (28.6)	0 (0.0)	
Severity of illness			
SAPS3	43.0 (30.5–54.5)	56.0 (46.5–58.0)	0.030
SOFA	6.0 (4.5–7.5)	6.0 (5.0–8.5)	0.445
Other pathogens identified from respiratory specimens			
Cytomegalovirus	3 (10.7)	10 (43.5)	0.011
Virus other than cytomegalovirus	4 (14.3)	3 (13.0)	1.000
Bacteria	3 (10.7)	7 (30.4)	0.078
Time to anti-PCP treatment, hours	64.5 (32.5–103.3)	46.2 (26.6–91.7)	0.447
Empiric treatment	16 (57.1)	15 (65.2)	0.765
Failure to initial treatment	6 (21.4)	16 (69.6)	0.002

*ICU* intensive care unit, *WBC* white blood cell, *CRP* C-reactive protein, *PaO*_*2*_*/FiO*_*2*_ arterial partial pressure of oxygen/fraction of inspired oxygen, *D(A-a)O*_*2*_ alveolar-arterial oxygen gradient, *SAPS 3* Simplified Acute Physiology Score 3, *SOFA* Sequential Organ Failure Assessment, *PCP Pneumocystis* pneumonia

**Table 5:** Clinical factors affecting hospital mortality

	Univariable			Multivariable^†^		
	Crude OR	95% CI	*P* value	Adjusted OR	95% CI	*P* value
Age	1.06	1.01–1.10	0.011	1.07	1.01–1.14	0.034
Gender	0.43	0.12–1.50	0.184	0.80	0.10–6.06	0.826
SAPS 3	1.05	1.00–1.10	0.033	1.05	0.98–1.13	0.149
Serum albumin, g/dL	0.47	0.18–1.26	0.133	0.58	0.13–2.56	0.469
Cytomegalovirus co-infection	5.50	1.44–20.96	0.013	3.19	0.52–19.63	0.212
Bacterial co-infection	3.65	0.82–16.19	0.089	2.90	0.34–24.32	0.327
Time to anti-PCP treatment, hour	1.00	0.99–1.01	0.939	1.01	0.99–1.02	0.307
Failure to initial treatment	8.38	2.36–29.74	0.001	14.12	2.23–89.38	0.005

*OR* odds ratio, *CI* confidence interval, *SAPS* Simplified Acute Physiology Score 3, *PCP Pneumocystis* pneumonia

## Discussion

This study investigated the association between the time to anti-PCP treatment and clinical outcomes in HIV-negative patients with PCP who presented with hypoxemic respiratory failure. Our results suggest that the clinical outcomes in HIV-negative patients with PCP and respiratory failure are unrelated to the time to initiation of anti-PCP treatment. In addition, clinical outcomes of patients received early empiric treatment were not better than those of patients received definitive treatment after microbiologic diagnosis.

Over the last decade, there has been a substantial decline in PCP-related mortality among HIV-positive patients, while there is increasing mortality (from PCP) in HIV-negative patients [9, 10]. Delay in therapy in HIV-negative patients compared to HIV-positive patients was associated with higher mortality [16, 17]. This finding suggests that empiric therapy for PCP should be initiated in patients with high clinical suspicion for PCP. Unfortunately, there is no clinical tool that rapidly identifies patients at risk of PCP in whom empiric treatment is warranted. In addition, the prevalence of PCP in HIV-negative cancer patients with acute respiratory failure and diffuse pulmonary infiltrates is < 10% [30, 31]. Empiric therapy for PCP in most patients, therefore, would be unnecessary and potentially cause harm.

Although several prior studies found that delayed therapy in anti-PCP treatment was associated with higher mortality [16, 17], we found no such difference. It is difficult to explain this discrepancy. It may be due to the difference in the units that were used to measure delayed treatment (hours vs. days). Unlike in previous studies, we measured the time to anti-PCP treatment was calculated in hours instead of days [32]. Another potential explanation for these conflicting results is differences in the study populations. We only included HIV-negative patients in our analysis of the association of time to anti-PCP treatment on mortality. In addition, the majority of patients required mechanical ventilation on admission. These results suggest against empiric treatment PCP in HIV-negative patients who present with hypoxemic respiratory failure given the various (potentially harmful) side effects of this treatment.

PCP in HIV-negative patients causes acute fulminant pneumonia with abrupt-onset respiratory failure and the need for mechanical ventilation [9, 10]. We observed a high mortality rate in our patients who required mechanical ventilation, which was consistent with the findings from previous reports [14–16, 18]. In addition, the severity of illness (at the time of ICU admission) was associated with higher mortality. These findings suggest that a patient’s general condition and underlying disease associated with PCP are more important than is early initiation of anti-PCP treatment in HIV-negative patients.

Given its high efficacy and bioavailavility, TMP/SMZ is used as a first-line agent in the treatment of PCP in patients with or without HIV infection [2, 9, 11]. Despite this, treatment failure has been reported with TMP/SMZ in 10–40% of patients [2]. Although large randomized trials have shown the efficacy of TMP/SMZ in HIV-positive patients, only a few small observational studies have demonstrated this in HIV-negative patients [9, 10]. Therefore, future studies must evaluate the potential association between failure of the initial treatment with TMP/SMX and increased mortality in HIV-negative patients. However, it might be difficult to distinguish the reasons for clinical deterioration, including progressive disease, adverse or failed treatment effects, or concomitant infection. In this study, all of our patients were initially treated with TMP/SMZ. Failure of the initial antimicrobial treatment for PCP was significantly associated with increased mortality. After adjusting for potentially confounding factors, failure of the initial antimicrobial treatment was still significantly associated with increased mortality. In most cases of treatment failure, however, the most likely cause of mortality was disease severity (before diagnosis and treatment), rather than drug resistance [33–35]. Regardless, future studies are needed to substantiate our findings with more patients.

There are several potential limitations to our study that should be acknowledged. First, given its retrospective observational design, this study may have been subject to selection bias. In addition, this study was conducted at a single center with a specialized ICU for a large number of cancer patients receiving chemotherapy. Therefore, our findings may not be readily generalizable to other institutions or patient populations. Finally, we could not compare the results with HIV-positive patients, since the number of HIV-positive patients diagnosed with PCP was too small to compare during the study period. However, patients with hematologic malignancies and solid tumors receiving chemotherapy do have a higher risk of PCP compared to that of other HIV-negative patients [6–8]. Therefore, our results represent actual practice in the treatment of PCP in HIV-negative patients.

## Conclusion

In conclusion, our data suggest that there is no relationship between the time to anti-PCP treatment and the treatment outcome in HIV-negative patients with PCP and associated hypoxic respiratory failure.

## Supplementary information

**Additional file 1.** **Table S1.** Comparison of baseline characteristics of 51 patients according to the quartiles of time to anti-PCP treatment.

**Additional file 2.** **Table S2.** Comparison of baseline patient characteristics according to initiation time of anti-PCP treatment.

**Additional file 3.** **Table S3.** Comparison of diagnosis and treatment of PCP and clinical outcomes according to initiation time of anti-PCP treatment.

## Data Availability

The data that support the findings of this study are available on request from the corresponding author. The data are not publicly available due to privacy or ethical restrictions.

## Abbreviations

BAL: Bronchoalveolar lavage
CI: Confidence interval
CMV: Cytomegalovirus
D(A-a)O_2_: Alveolar-arterial oxygen gradient
DNA: Deoxyribonucleic acid
ED: Emergency department
HIV: Human immunodeficiency virus
ICU: Intensive care unit
IQR: Interquartile range
OR: Odds ratio
PCP: *Pneumocystis* pneumonia
PCR: Polymerase chain reaction
PF: Arterial partial pressure of oxygen/fraction of inspired oxygen
SAPS 3: Simplified Acute Physiology Score 3
SOFA: Sequential Organ Failure Assessment
TBLB: Transbronchial lung biopsy
TMP/SMX: Trimethoprim/sulfamethoxazole
