# Supplementary material for: Association of time-to-treatment with outcomes of Pneumocystis pneumonia with respiratory failure in HIV-negative patients
Source: Respir Res. 2019 Sep 26;20:213. doi: 10.1186/s12931-019-1188-6 (PMC6761721; doi:10.1186/s12931-019-1188-6)
Supplement: Supplementary file 1 — Additional file 1. Table S1. Comparison of baseline characteristics of 51 patients according to the quartiles of time to anti-PCP treatment. [file 12931_2019_1188_MOESM1_ESM.docx]

**Supplement Table S1.** Comparison of baseline characteristics of 51 patients according to the quartiles of time to anti-PCP treatment.

| Characteristics | 1^st^ quartile (< 28.0 hours)  (n = 13) | 2^nd^ quartile (28.0 – 58.0 hours)  (n = 13) | 3^rd^ quartile (58.0 – 97.8 hours)  (n = 12) | 4^th^ quartile (≥97.8 hours)  (n = 13) | P value |
| --- | --- | --- | --- | --- | --- |
| Age, years | 52.0 (45.0 – 65.0) | 59.0 (52.0 – 67.0) | 57.5 (47.0 – 67.5) | 38.0 (34.0 – 52.0) | 0.150 |
| Gender, male | 8 (61.5) | 10 (76.9) | 7 (58.3) | 10 (76.9) | 0.631 |
| Underlying disease |  |  |  |  | 0.780 |
| Malignancy |  |  |  |  |  |
| Hematologic | 6 (46.2) | 6 (46.2) | 5 (41.7) | 4 (30.8) |  |
| Solid | 0 (0.0) | 1 (7.7) | 3 (25.0) | 3 (23.1) |  |
| Solid organ transplant | 5 (38.5) | 5 (38.5) | 3 (25.0) | 5 (38.5) |  |
| Others^*^ | 2 (15.4) | 1 (7.7) | 1 (8.3) | 1 (7.7) |  |
| Immunosuppressive agent use, previous month^†^ |  |  |  |  | 0.501 |
| Steroid only | 7 (53.8) | 4 (30.8) | 2 (16.7) | 5 (38.5) |  |
| Chemotherapy only | 7 (53.8) | 8 (61.5) | 7 (58.3) | 7 (53.8) |  |
| Steroid with chemotherapy | 0 (0.0) | 1 (7.7) | 2 (16.7) | 1 (7.7) |  |
| Prednisolone-equivalent dose, mg, if steroid used | 16.1 (3.5 – 28.3) | 20.1 (8.1 – 34.3) | 15.1 (1.2 – 42.8) | 14.4 (6.3 – 43.9) | 0.946 |
| Prophylaxis for pneumocystis | 1 (7.7) | 1 (7.7) | 0 (0.0) | 0 (0.0) | 0.572 |
| Chest radiography findings |  |  |  |  |  |
| Pleural effusion | 3 (23.1) | 2 (15.4) | 4 (33.3) | 2 (15.4) | 0.662 |
| Radiographic pulmonary pattern |  |  |  |  | 0.877 |
| Focal or diffuse alveolar pattern | 5 (38.5) | 6 (46.2) | 5 (41.7) | 8 (61.5) |  |
| Focal or diffuse interstitial pattern | 1 (7.7) | 2 (15.4) | 1 (8.3) | 1 (7.7) |  |
| Focal or diffuse alveolar-interstitial pattern | 7 (53.8) | 5 (38.5) | 6 (50.0) | 4 (30.8) |  |
| Laboratory findings on ICU admission |  |  |  |  |  |
| WBC blood cells,/μL | 13240 (9300 – 17890) | 9540 (7670 – 11400) | 5560 (2980 – 7860) | 5390 (3000 – 9130) | 0.012^a^ |
| Neutrophils,/μL | 11100 (5790 – 16550) | 7440 (5392 – 9500) | 4680 (2325 – 6060) | 3600 (1990 – 5500) | 0.027^a^ |
| Lymphocytes,/μL | 680 (338 – 1330) | 770 (410 – 1280) | 452 (250 – 730) | 530 (350 – 720) | 0.338 |
| Albumin,g/dL | 3.2 (2.5 – 3.6) | 3.0 (2.7 – 3.3) | 2.8 (2.5 – 3.3) | 2.4 (2.2 – 3.0) | 0.301 |
| CRP, mg/dL | 13.7 (8.3 – 18.1) | 12.4 (6.4 – 19.7) | 9.1 (7.4 – 19.1) | 18.8 (11.2 – 20.6) | 0.719 |
| PaO_2_/FiO_2_ ratio, mmHg | 148.5 (128.0 – 174.0) | 146.4 (122.8 – 158.6) | 132.8 (93.6 – 163.1) | 115.8 (91.2 – 164.8) | 0.524 |
| D(A-a)O_2_, mmHg | 37.5 (29.9 – 41.6) | 49.6 (38.8 – 62.7) | 47.1 (35.0 – 56.8) | 31.4 (15.8 – 44.9) | 0.021^a^ |
| Organ failures on ICU admission |  |  |  |  |  |
| Shock | 2 (15.4) | 3 (23.1) | 3 (25.0) | 4 (30.8) | 0.832 |
| Renal failure requiring RRT | 0 (0.0) | 0 (0.0) | 0 (0.0) | 1 (7.7) | 0.394 |
| Respiratory support on ICU admission day |  |  |  |  |  |
| Mechanical ventilation | 11 (84.6) | 11 (84.6) | 10 (83.3) | 11 (84.6) | 0.948 |
| High-flow nasal cannula | 1 (7.7) | 3 (23.1) | 2 (16.7) | 2 (15.4) | 0.759 |
| Severity of illness |  |  |  |  |  |
| SAPS3 | 50.0 (42.0 – 56.0) | 43.0 (30.0 – 56.0) | 57.5 (47.0 – 64.5) | 40.0 (33.0 – 53.0) | 0.029^b^ |
| SOFA | 6.0 (5.0 – 7.0) | 6.0 (3.0 – 7.0) | 6.0 (5.0 – 9.5) | 6.0 (4.0 – 8.0) | 0.597 |

ICU, intensive care unit; WBC, white blood cell; CRP, C-reactive protein; PaO_2_/FiO_2,_ arterial partial pressure of oxygen/fraction of inspired oxygen; [D(A-a)O_2_], alveolar-arterial oxygen gradient; RRT, renal replacement therapy; SAPS 3, Simplified Acute Physiology Score 3; SOFA, Sequential Organ Failure Assessment.

^*^Others include 2 glomerulonephritis, 2 interstitial lung disease, and 1 liver cirrhosis.

^†^Chemotherapy includes 4 patients with T-cell immunosuppressant.

^a^, *P* < 0.05 in the post hoc analysis between 1^st^ quartile group and 4^th^ quartile group.

^b^, *P* < 0.05 in the post hoc analysis between 3^rd^ quartile group and 4^th^ quartile group.
