# Supplementary material for: Association of time-to-treatment with outcomes of Pneumocystis pneumonia with respiratory failure in HIV-negative patients
Source: Respir Res. 2019 Sep 26;20:213. doi: 10.1186/s12931-019-1188-6 (PMC6761721; doi:10.1186/s12931-019-1188-6)
Supplement: Supplementary file 2 — Additional file 2. Table S2. Comparison of baseline patient characteristics according to initiation time of anti-PCP treatment. [file 12931_2019_1188_MOESM2_ESM.docx]

**Supplement Table S2.** Comparison of baseline patient characteristics according to initiation time of anti-PCP treatment

| Characteristics | Early empiric treatment (n = 31) | Definitive treatment (n = 20) | P value |
| --- | --- | --- | --- |
| Age, years | 54.0 (48.0 – 64.5] | 42.0 [34.5 – 67.5] | 0.150 |
| Gender, male | 23 (74.2) | 12 (60.0) | 0.449 |
| Underlying disease  Malignancy  Hematologic  Solid  Solid organ transplant  Kidney  Liver  Heart  Lung  Others^*^ | 14 (45.2)  11  3  14 (45.2)  7  4  2  1  3 (9.7) | 14 (70.0)  10  4  4 (20.0)  4  0  0  0  2 (10.0) | 0.146  0.290  1.000 |
| Immunosuppressive agent use, previous month^†^  Steroid only  Chemotherapy only  Steroid with chemotherapy | 13 (41.9)  1 (3.2)  17 (54.8) | 5 (25.0)  3 (15.0)  12 (60.0) | 0.196 |
| Prednisolone-equivalent dose, mg, if steroid used | 19.3 [8.8 – 32.7] | 12.6 [3.1 – 43.1] | 0.569 |
| Prophylaxis for pneumocystis | 2 (6.5) | 0 (0.0) | 0.674 |
| Chest radiography findings  Pleural effusion  Radiographic pulmonary pattern  Focal or diffuse alveolar pattern  Focal or diffuse interstitial pattern  Focal or diffuse alveolar-interstitial pattern | 9 (29.0)  14 (45.2)  2 (6.5)  15 (48.4) | 2 (10.0)  10 (50.0)  3 (15.0)  7 (35.0) | 0.206  0.479 |
| Laboratory findings on ICU admission  WBC blood cells,/μL  Neutrophils,/μL  Lymphocytes,/μL  Albumin,g/dL  CRP, mg/dL  PaO_2_/FiO_2_ ratio, mmHg  D(A-a)O_2_, mmHg | 10180 (5925 – 14770)  7980 (4090 – 11720)  560 (300 – 1068)  3.0 (2.5 – 3.5)  9.3 (7.4 – 19.1)  144.8 (116.9 – 167.0)  39.9 (32.3 – 53.7) | 5925 (3665 – 8445)  4555 (2880 – 7095)  590 (344 – 1020)  2.5 (2.3 – 3.0)  16.5 (8.5 – 22.6)  135.6 (102.3 – 163.1)  38.9 (24.2 – 51.5) | 0.021  0.120  0.885  0.075  0.171  0.653  0.534 |
| Organ failures on ICU admission  Shock  Renal failure requiring renal replacement therapy | 6 (19.4)  0 (0.0) | 6 (30.0)  1 (5.0) | 0.591  0.823 |
| Respiratory support on ICU admission day  Mechanical ventilation  High-flow nasal cannula | 26 (83.9)  5 (16.1) | 17 (85.0)  3 (15.0) | 1.000 |
| Severity of illness  SAPS 3  SOFA | 50.0 (39.0 – 58.0)  6.0 (4.5 – 7.0) | 45.0 (35.5 – 56.5)  6.5 (5.5 – 9.0) | 0.451  0.276 |

Data are presented as number (percentage) or as median (interquartile range).

PCP, *Pneumocystis* pneumonia; ICU, intensive care unit; WBC, white blood cell; CRP, C–reactive protein; PaO_2_/FiO_2,_ arterial partial pressure of oxygen/fraction of inspired oxygen; [D(A–a)O_2_], alveolar–arterial oxygen gradient; SAPS 3, Simplified Acute Physiology Score 3; SOFA, Sequential Organ Failure Assessment.

^*^Others include 2 glomerulonephritis, 2 interstitial lung disease, and 1 liver cirrhosis.

^†^Chemotherapy includes 4 patients with T–cell immunosuppressant.
