# Supplementary material for: Association of time-to-treatment with outcomes of Pneumocystis pneumonia with respiratory failure in HIV-negative patients
Source: Respir Res. 2019 Sep 26;20:213. doi: 10.1186/s12931-019-1188-6 (PMC6761721; doi:10.1186/s12931-019-1188-6)
Supplement: Supplementary file 3 — Additional file 3. Table S3. Comparison of diagnosis and treatment of PCP and clinical outcomes according to initiation time of anti-PCP treatment. [file 12931_2019_1188_MOESM3_ESM.docx]

**Supplement Table S3.** Comparison of diagnosis and treatment of PCP and clinical outcomes according to initiation time of anti-PCP treatment

| Characteristics | Early empiric treatment (n = 31) | Definitive treatment (n = 20) | P value |
| --- | --- | --- | --- |
| Microbiological diagnosis  Bronchoalveolar lavage fluid  Lung biopsy specimen^*^ | 24 (77.4)  7 (22.6) | 15 (75.0)  5 (25.0) | 0.450 |
| Time to anti–PCP treatment, hours | 34.2 (18.4 – 66.7) | 91.7 (54.0 – 111.0) | < 0.001 |
| Other pathogens identified from respiratory specimens  Cytomegalovirus  Virus other than cytomegalovirus^†^  Bacteria  MRSA  *Acinetobacter*  *Pseudomonas* | 9 (29.0)  6 (19.4)  3 (9.7)  4 (12.9)  2 (6.5) | 6 (30.0)  1 (5.0)  2 (10.0)  0 (0.0)  0 (0.0) | 1.000  0.223  1.000  0.254  0.674 |
| Initial treatment regimen  Trimethoprim/sulfamethoxazole | 31 (100.0) | 20 (100.0) | NA |
| Adjunctive corticosteroid treatment | 30 (96.8) | 20 (100.0) | 1.000 |
| Failure to initial treatment | 15 (48.4) | 7 (35.0) | 0.514 |

Data are presented as number (percentage) or as median (interquartile range).

PCP, *Pneumocystis* pneumonia; MRSA, methicillin–resistant *Staphylococcus aureus*.

^*^7 patients had positive results for the presence of *P.jirovecii* in both BAL fluid and biopsy specimens.

^†^Viruses other than cytomegalovirus include rhinovirus (n=3), coronavirus (n=2), and rhinovirus (n=2).
